# Our New York Letter

**Published:** 1888-06

**Authors:** 


### Correspondence.

### OUR NEW YORK LETTER.

DEATH'S VICTIMS IN NEW YORK—MR. CONKLING—DR. CORNELIUS REA AGNEW—SUDDEN DEATH OF DR. LORING—THIEVING MEDICAL STUDENTS—SAMUEL J. TILDEN'S PHYSICIAN SUING FOR HIS BILL—RESIGNATION OF DR. HENRY B. SANDS—HOSPITAL CHANGES.

*To Editors Atlanta Medical and Surgical Journal:*

During the past few weeks several instances of sickness and death among our most notable men have followed closely upon each other. Dr. Wm. A. Hammond has been seriously ill from an abscess, occupying the side of the face and neck, for which he has been under the care of Drs. Satterthwaite and Abbé. Incisions with drainage have been resorted to, and at last accounts his condition was very favorable. Our distinguished townsman, Senator Roscoe Conkling, was taken sick with what at first was supposed to be a simple boil in the ear. Dr. Fordyce Barker was called and found the patient suffering from otitis media, and as operative interference was considered necessary, Dr. Sands was also requested to assist in the case. In spite of treatment, however, purulent pachymeningitis developed and death occurred several days later. Mr. Conkling was in poor general condition at the beginning of the attack, and this probably contributed to the disease running such an unfavorable course.

Dr. Cornelius R. Agnew was taken sick while actively engaged in professional duties and died in about one week. He was attacked first with perityphilitis, which was followed by a perforation of the vermiform appendix and the formation of a peritoneal abscess. An operation was performed and the pus evacuated, but unfortunately diffuse peritonitis set in, which was rapidly fatal. Drs. Delafield and Sands were the medical attendants. Cornelius Rea Agnew was born in New York on the 8th of August, 1830. After a thorough medical education here, he went abroad and continued to study under the best teachers of Europe, returning later to this city, where he began the practice of ophthalmology and otology. His subsequent career was an eventful and illustrious one. He became a member of the United States Sanitary Commission, and in 1858 he was appointed Surgeon-General of the State of New York. In 1866 he organized the ophthalmic clinic at the College of Physicians and Surgeons, and later he established the Manhattan Eye and Ear Hospital and several others. He was connected with numerous medical and educational institutions and with all the leading medical and scientific societies. During his thirty years of work as an oculist and aurist, he acquired a world-wide reputation and contributed many useful articles on the diseases of the eye and ear. Dr. Agnew was considered a man who united superior intelligence and profound learning with the most noble traits of character, and his loss is certainly an irreparable one. Only a few days elapsed after Dr. Agnew's funeral when the sudden death of Dr. Edward G. Loring occurred. He had complained for a couple of days of a slight pain in the chest, but otherwise appeared perfectly well. Leaving his home in the afternoon, he visited his yacht in the East River, and while returning through 74th street suddenly fell at the corner of Park avenue. Dr. H. B. Conrad, who resides near here, immediately went to his assistance, but found that life was extinct. Strange as it may seem, the autopsy made by the coroner's physician failed to reveal any apparent cause for this very sudden death. Dr. Loring was a graduate of the Harvard Medical School and was about forty-eight years of age. As an ophthalmologist he ranked among the best and his name was mentioned as a fit successor of Dr. Agnew in the College of Physicians and Surgeons. Although not much of an operator, he excelled in all other departments of his specialty, and his work on the

Ophthalmoscope, which had just been published, is considered his best effort among his many literary contributions. At one time he was a partner with Dr. Agnew during a period of six years, after which he built up an independent practice and was actively engaged in the New York Eye and Ear Infirmary up to the date of his death.

Sometime ago quite a quantity of medicines was stolen from Bellevue Hospital, and recently instruments and other apparatus of value have been disappearing from the Loomis Laboratory. The attention of the police was called to the matter, and after an investigation they succeeded in finding a portion of the missing articles in the possession of two graduates of the college. Nearly all the stolen property, amounting to about $1,000 worth, has been returned. It is said that one of the guilty parties only graduated a few weeks ago and the other was connected with the dispensary, but the college faculty are not inclined to have them prosecuted.

Dr. Charles E. Simmons, of this city, has begun suit against the executors of the estate of the late Samuel J. Tilden to recover the amount of his bill for professional services rendered to the former candidate for the presidency. Dr. Simmons says that he was in almost constant attendance upon Mr. Tilden during his protracted sickness, and when the claims against the estate were being liquidated, he sent in his bill for the modest sum of $143,000. The executors, however, have refused to pay the doctor, and have demanded that a bill of particulars be sent in explaining just what professional services have been rendered. The doctor is perfectly willing to do this, and the case promises to be interesting, but it will probably not come off before fall.

Dr. Henry B. Sands, who for a number of years has had a continuous surgical service at the Roosevelt Hospital, has resigned this position. The operative work here has been very arduous, and I understand that it is his intention to take life a little more leisurely and do mostly a consulting practice. Dr. Sands is probably unequalled in New York as a surgeon, and his hospital vacancy has been filled by Dr. Charles McBurney.

Dr. Lewis A. Stimson has been appointed visiting surgeon to the New York and also to the Chambers Street Hospital, and Dr. William T. Bull has resigned his position in the last-named institution. A. F.

May 8th, 1888.

